# Inhibition of the HMGB1-RAGE Axis Attenuates Microglial Inflammation and Ameliorates Hypoxia-Induced Cognitive Impairment

**DOI:** 10.3390/ijms26188782

**Published:** 2025-09-09

**Authors:** Chenlin Liu, Haowei Zhang, Ruili Guan, Yuankang Zou, Mengyu Chen, Mingrui Du, Wenjing Luo, Jianbin Zhang

**Affiliations:** Department of Occupational and Environmental Health, The Ministry of Education Key Lab of Hazard Assessment and Control in Special Operational Environment, School of Public Health, The Fourth Military Medical University, Xi’an 710032, China; liuchenlin@fmmu.edu.cn (C.L.); m18909258023@163.com (H.Z.); guanruili@fmmu.edu.cn (R.G.); sydzouyk@163.com (Y.Z.); 18394010154@163.com (M.C.); dmr12345@fmmu.edu.cn (M.D.)

**Keywords:** high-altitude hypobaric hypoxia (HAHH), microglia, RAGE, HMGB1, inflammation, cognitive function

## Abstract

The mechanisms underlying the abnormal activation of microglia affecting cognitive function under high-altitude hypobaric hypoxia (HAHH) have not been fully elucidated. This study aims to investigate the effects of HAHH on the expression of the receptor for advanced glycation end-products (RAGE) in hippocampal microglia of mice and to explore the role of RAGE inhibitors in alleviating HAHH-induced microglial inflammation and cognitive impairment. Mice were exposed to HAHH via a multi-environment simulation chamber, and RNA sequencing, qPCR, WB, flow cytometry and immunohistochemistry showed that HAHH exposome significantly increased RAGE expression in hippocampal microglia of mice (*p* < 0.001 vs. normoxia), which was closely related to microglial neuroinflammatory responses. RAGE inhibitor (FPS-ZM1) alleviated HAHH-induced microglial inflammation (TNF-α decreased by 64%, *p* < 0.001; CD86^+^ cells decreased by 42%, *p* < 0.001) and improved cognitive function in mice (Y-maze novel arm time: 28.08 ± 5.14 s vs. hypoxia 19.67 ± 4.68 s, *p* = 0.016; NORT recognition index: 0.52 ± 0.05 vs. hypoxia 0.33 ± 0.07, *p* < 0.001). Mechanistic studies revealed that RAGE inhibitors reduced microglial inflammation by inhibiting the MAPK pathway and decreasing nuclear translocation of NF-κB p65. Furthermore, high-mobility group box 1 (HMGB1) expression increased under hypoxic conditions (*p* < 0.001 vs. normoxia) and positively regulated RAGE expression. HMGB1 inhibitors reduced RAGE expression and attenuated HAHH-induced microglial inflammation. Overall, the HAHH exposome induces microglial inflammation via the HMGB1-RAGE-NF-κB pathway. RAGE and HMGB1 inhibitors may serve as novel therapeutic strategies to mitigate HAHH-induced cognitive impairment, providing a theoretical basis for the treatment of cognitive impairment.

## 1. Introduction

High-altitude hypobaric hypoxia (HAHH) has garnered increasing attention for its impact on central nervous system (CNS) function [1]. Studies have shown that prolonged hypoxia exposome can lead to neurotoxicity, which may be associated with oxidative stress, metabolic disturbances, and neuroinflammatory responses [2,3,4]. Microglia, the brain’s resident immune cells, have a significant impact on the development of inflammation [5,6]. Under hypoxic conditions, dysregulated microglial activation may contribute to neuroinflammatory responses, thereby affecting cognitive function [7,8]. While transient oxygen-glucose deprivation (OGD) or hypoxic–ischemic brain damage (HIBD) studies focus on immediate microglial cell death pathways [9,10], high-altitude hypobaric hypoxia reveals how chronic microglial inflammation drives progressive cognitive impairment—a process more relevant to altitude-related neurodegeneration than stroke. However, the mechanisms underlying the abnormal activation of microglia under HAHH have not been fully elucidated. Therefore, revealing and modulating these mechanisms might serve as an effective strategy to counteract cognitive deficits resulting from HAHH exposome and promote functional recovery.

RAGE, a transmembrane receptor belonging to the immunoglobulin superfamily, contributes to various pathological processes, including diabetes, neurodegenerative diseases, and inflammatory responses [11,12,13]. In this study, RNA sequencing revealed differences in the AGE-RAGE signaling pathway following HAHH exposome, which is closely related to microglial activation. Recent studies confirm HMGB1-RAGE as a master regulator of neuroinflammation in acute injury models [9] and neurodegeneration [14]. However, the effects of RAGE on chronic microglial inflammation and cognitive function under HAHH remain unclear. Here, we characterize HAHH-induced alterations in RAGE expression patterns within hippocampal microglial populations of mice and further investigate the role of RAGE inhibitors in alleviating HAHH-induced microglial inflammation and cognitive impairment.

HMGB1, a damage-associated molecular pattern (DAMP) molecule, is upregulated under various stress conditions, including hypoxia exposome [14,15]. HMGB1 contributes to the pathogenesis of multiple CNS disorders through its role in neurotoxicity, including traumatic brain injury, stroke, and multiple sclerosis [16,17,18]. RAGE, as a receptor for HMGB1, can bind to it and influence inflammatory and immune responses [19,20,21,22]. Whether HMGB1 acts upstream of RAGE and whether the HMGB1-RAGE axis plays a key regulatory role in HAHH-induced inflammation and cognitive impairment remain unclear.

Therefore, we hypothesized that HAHH activates the HMGB1-RAGE axis in hippocampal microglia, driving neuroinflammation and cognitive impairment—a process reversible by RAGE inhibition. In this study, our objective is to confirm whether the HMGB1-RAGE axis is linked to HAHH-induced inflammation and cognitive impairment and to assess therapeutic efficacy of inhibition of the HMGB1-RAGE axis.

## 2. Results

### 2.1. HAHH Increases RAGE Expression in Mouse Hippocampal Microglia

**Figure 1:**
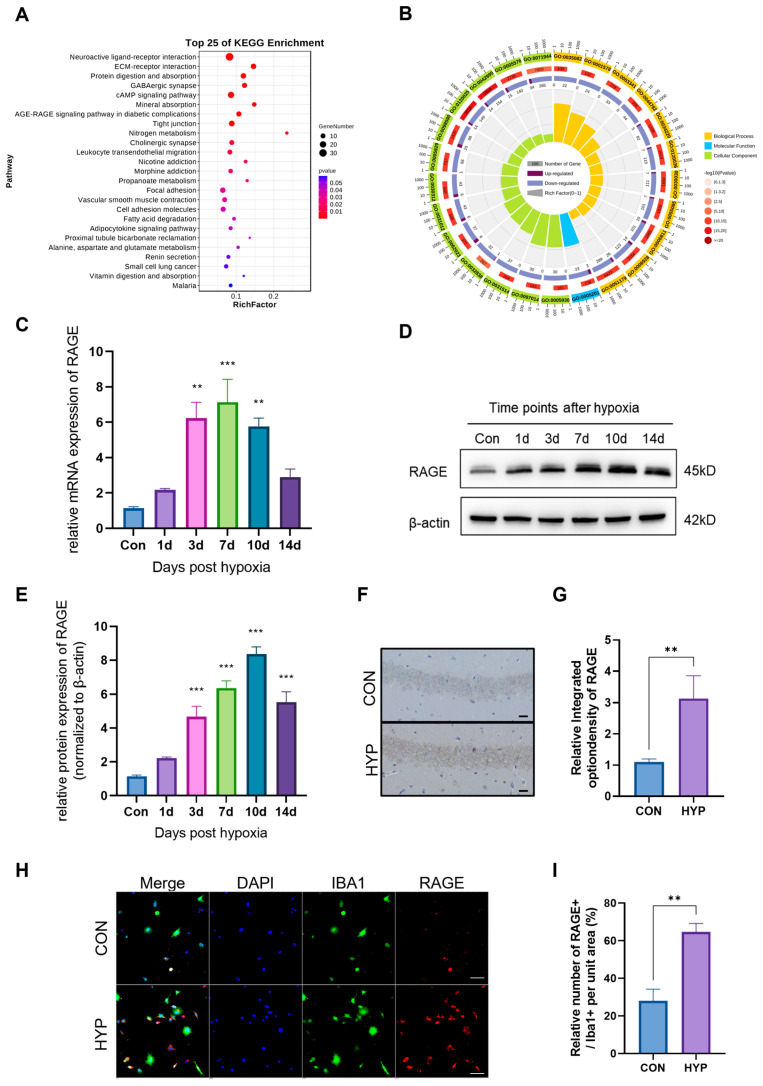
The effects of HAHH exposome on RAGE expression in the mouse hippocampus. (**A**) KEGG enrichment analysis of differentially expressed genes in the hippocampus after HAHH exposome. (**B**) GO enrichment analysis of differentially expressed genes in the hippocampus after HAHH exposome. (**C**) qPCR analysis of RAGE mRNA levels in the hippocampus of mice exposed to HAHH over time. Data are presented as fold changes relative to normoxic controls. *n* = 3, analyzed by Student’s *t*-test for two-group comparisons. (**D**) WB analysis of RAGE protein expression in the hippocampus of mice exposed to HAHH over time. (**E**) Quantitative analysis of RAGE protein expression in the hippocampus of mice exposed to HAHH over time. Data are presented as relative densitometry units normalized to β-actin. *n* = 3, analyzed by Student’s *t*-test for two-group comparisons. (**F**) Representative images of immunohistochemical staining for RAGE in the hippocampus of mice after HAHH exposome. Scale bar = 50 μm. (**G**) Quantitative analysis of RAGE immunohistochemical staining in the hippocampus of mice after HAHH exposome, expressed as mean optical density. *n* = 3, analyzed by Student’s *t*-test for two-group comparisons. (**H**) Immunofluorescence confocal microscopy of primary microglia isolated from the hippocampus of HAHH-exposed mice with staining for RAGE (FITC-conjugated, green), IBA1 (Cy3-labeled, red) and DAPI for nuclei (blue). Scale bar = 20 μm. Data from *n* = 3/group, analyzed by Student’s *t*-test for two-group comparisons. (**I**) Quantitative analysis of RAGE-positive cells as a percentage of IBA1-positive microglia in the hippocampus of HAHH-exposed mice. *n* = 3, analyzed by Student’s *t*-test for two-group comparisons. ** *p* < 0.01, *** *p* < 0.001.

In our study, a multi-environment simulation chamber was used to simulate a hypoxic environment at an altitude of 6000 m. Subsequently, we performed RNA sequencing on the hippocampus of HAHH-exposed mice models. KEGG and GO enrichment analyses of differentially expressed genes showed differences in pathways related to neuro- and synaptic functions, signal transduction, and energy metabolism, which have been previously associated with HAHH exposome, further confirming the reliability and credibility of the model. Intriguingly, our investigation revealed that the AGE-RAGE signaling pathway, which is involved in diabetic complications, also showed significant differences following HAHH exposome and ranked high among the pathways (Figure 1A,B). Therefore, we further isolated the hippocampus from HAHH-exposed mice to examine the changes in RAGE expression. qPCR data showed that the mRNA levels of RAGE significantly increased from 1 to 14 days of HAHH exposome (Figure 1C). WB (WB) also demonstrated increased protein expression of RAGE following HAHH exposome (Figure 1D,E). Similarly, we used immunohistochemistry on brain sections to detect RAGE expression levels, and our data indicated that RAGE expression was upregulated in the hippocampus of HAHH-exposed mice (*p* < 0.01 vs. normoxia) (Figure 1F,G). Considering that studies have confirmed a close correlation between RAGE expression and microglial neuroinflammatory responses, co-localization studies via dual immunofluorescence were conducted to map RAGE distribution relative to microglia (Figure 1H). Immunofluorescence showed that RAGE-positive staining mainly co-localized with IBA1-positive microglia following HAHH exposome (*p* < 0.01 vs. normoxia) (Figure 1I). RAGE was predominantly expressed on the microglial membrane, and the relative fluorescence density of RAGE in the hippocampus of HAHH-exposed mice increased, which also confirmed the upregulation of RAGE expression following HAHH exposome.

### 2.2. RAGE Inhibitor Attenuates HAHH-Induced Microglial Inflammation in the Mouse Hippocampus

**Figure 2:**
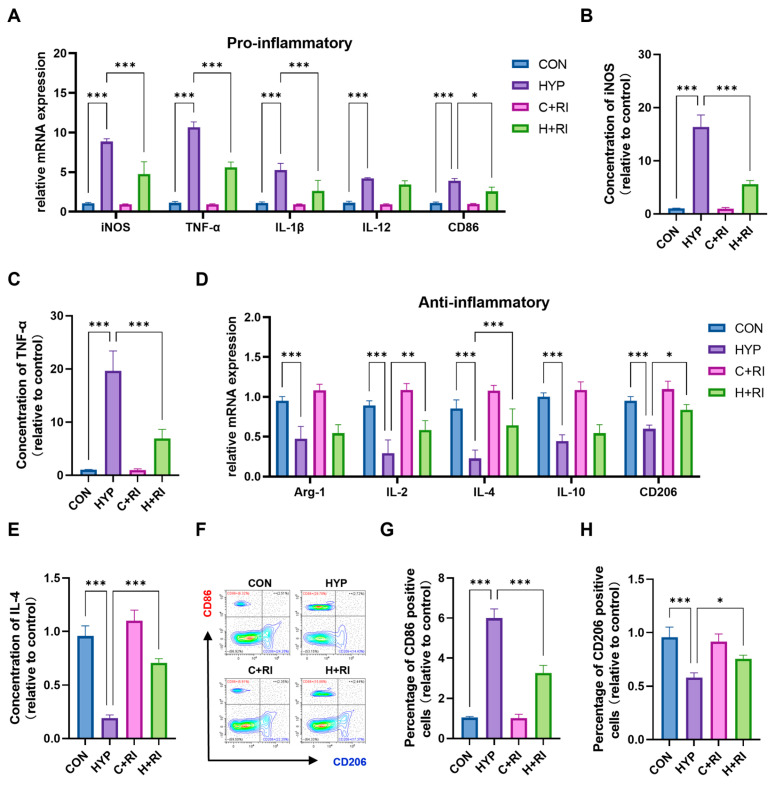
Effects of a RAGE inhibitor on HAHH-induced microglial inflammation. (**A**) qPCR analysis of pro-inflammatory markers (iNOS, TNF-α, IL-1β, IL-12, CD86) in microglial cells treated with RAGE inhibitor (FPS-ZM1) under hypoxic conditions. Data are presented as fold changes relative to normoxic controls. (**B**,**C**) ELISA analysis of iNOS and TNF-α levels in microglial cells treated with RAGE inhibitor under hypoxic conditions. (**D**) qPCR analysis of anti-inflammatory markers (Arg-1, IL-2, IL-4, IL-10, CD206) in microglial cells treated with RAGE inhibitor under hypoxic conditions. Data are presented as fold changes relative to normoxic controls. (**E**) ELISA analysis of IL-4 protein levels in microglial cells treated with RAGE inhibitor under hypoxic conditions. (**F**–**H**) Flow cytometry analysis of CD86-positive (red) and CD206-positive (blue) microglial cells in the hippocampus of mice treated with RAGE inhibitor under hypoxic conditions. Data are presented as fold changes relative to control group. *n* = 3, analyzed by one-way ANOVA with Tukey post hoc test for multiple comparisons. * *p* < 0.05, ** *p* < 0.01, *** *p* < 0.001.

To further explore RAGE’s impact on microglial activation, we quantified mRNA expression of pro- and anti-inflammatory markers in primary microglia isolated from hypoxic mice treated with the RAGE antagonist FPS-ZM1. Hypoxic conditions robustly increased transcript levels of key pro-inflammatory mediators: iNOS (~9.2-fold, *p* < 0.001), TNF-α (~12.3-fold, *p* < 0.001), IL-1β (~5.5-fold, *p* < 0.001), IL-12 (~4.1-fold, *p* < 0.001), and CD86 (~3.8-fold, *p* < 0.001) (Figure 2A). FPS-ZM1 administration significantly attenuated these increases for iNOS (reduced by 52%, *p* < 0.001 vs. HYP), TNF-α (48% reduction, *p* < 0.001), IL-1β (65% decrease, *p* < 0.001), and CD86 (30% suppression, *p* < 0.05), but failed to reverse IL-12 upregulation (*p* = 0.12 vs. HYP). ELISA confirmed elevated protein concentrations of iNOS (16.8-fold increase, *p* < 0.001) and TNF-α (18.4-fold, *p* < 0.001) in hypoxic microglia, which FPS-ZM1 reduced to near-baseline levels (iNOS: 79% reduction, *p* < 0.001; TNF-α: 64% decrease, *p* < 0.001) (Figure 2B,C).

Simultaneously, we conducted the parallel analysis of anti-inflammatory markers. We observed that the HAHH exposome led to suppression of Arg-1 (~0.45-fold, *p* < 0.001), IL-2 (~0.32-fold, *p* < 0.001), IL-4 (~0.25-fold, *p* < 0.001), IL-10 (~0.42-fold, *p* < 0.001), and CD206 (~0.43-fold, *p* < 0.001) mRNA (Figure 2D,E). FPS-ZM1 treatment significantly rescued expression of IL-2 (2.1-fold increase vs. HYP, *p* < 0.01), IL-4 (2.5-fold, *p* < 0.001), and CD206 (1.3-fold, *p* < 0.05) but showed limited efficacy on Arg-1 (*p* = 0.27) and IL-10 (*p* = 0.29).

Flow cytometric evaluation of microglial populations demonstrated HAHH-induced expansion of CD86^+^ cells and contraction of CD206^+^ populations (Figure 2F). In mice treated with the RAGE inhibitor, the number of CD86-positive microglia in the hippocampus significantly decreased, while the number of CD206-positive microglia significantly increased. FPS-ZM1 treatment effectively normalized these shifts in hippocampal microglia, reducing CD86^+^ by 42% (*p* < 0.001 vs. HYP) while augmenting CD206^+^ subsets 1.2-fold (*p* < 0.05), effectively restoring baseline polarization balance (Figure 2G,H). This demonstrated that the RAGE inhibitor restored most of the microglial inflammation caused by HAHH exposome. The above results prove that RAGE inhibition can reduce HAHH-induced microglial inflammation in the mouse hippocampus.

### 2.3. RAGE Inhibitors Restore Inflammation and Cognitive Impairment Induced by the HAHH Exposome in Mice

**Figure 3:**
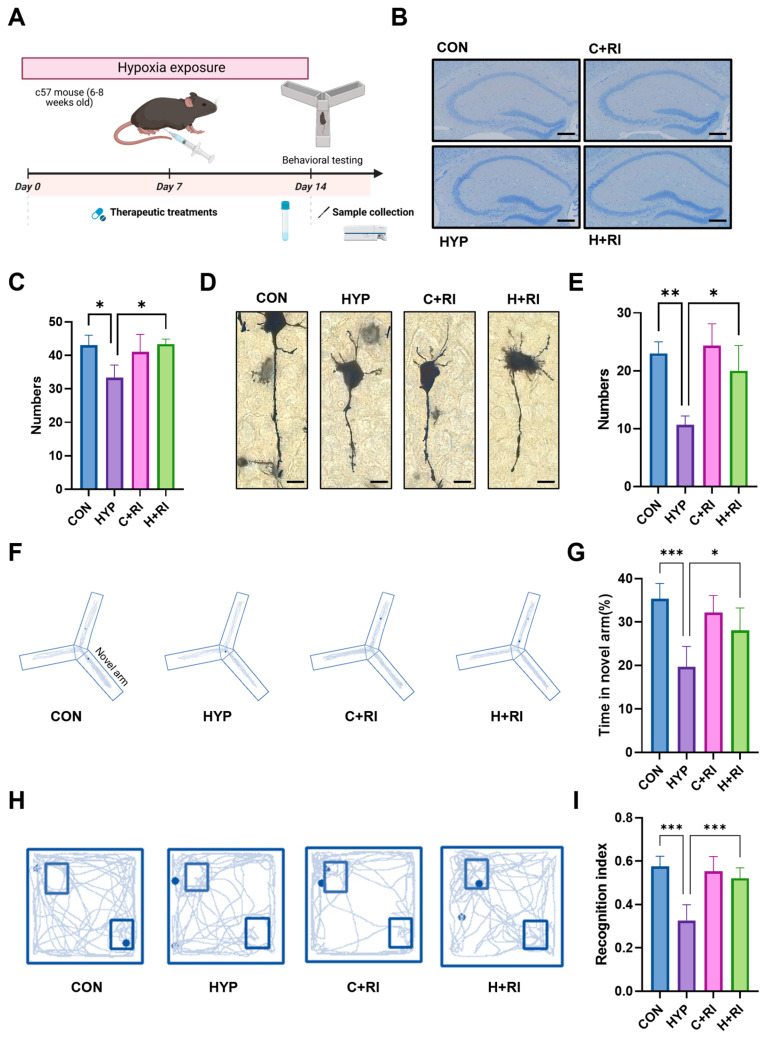
Effects of a RAGE inhibitor on HAHH-induced inflammation. (**A**) Schematic of chronic HAHH exposure, pharmacological intervention, behavioral testing, and molecular analysis timeline. (**B**) Representative images of Nissl staining of hippocampal neurons in mice treated with RAGE inhibitor under hypoxic conditions. Scale bar = 200 μm. (**C**) Quantitative analysis of neuronal density in hippocampus of mice treated with RAGE inhibitor under hypoxic conditions. (**D**) Representative images of Golgi staining of dendritic spines in hippocampus of mice treated with RAGE inhibitor under hypoxic conditions. Scale bar = 10 μm. (**E**) Comparative spine density measurements in hippocampus of mice treated with RAGE inhibitor under hypoxic conditions. (**F**) Representative images of Y-maze exploration trajectories in mice treated with RAGE inhibitor under hypoxic conditions. (**G**) Quantitative analysis of time spent exploring novel arm in Y-maze test in mice treated with RAGE inhibitor under hypoxic conditions. (**H**) Representative images of exploration trajectories in novel object recognition test (NORT) in mice treated with RAGE inhibitor under hypoxic conditions. (**I**) Quantitative analysis of recognition index in NORT in mice treated with RAGE inhibitor under hypoxic conditions. *n* = 12, analyzed by one-way ANOVA with Tukey post hoc test for multiple comparisons. * *p* < 0.05, ** *p* < 0.01, *** *p* < 0.001.

Microglial inflammation-mediated harmful effects are directly correlated with the extent of cognitive deterioration secondary to HAHH exposome. Considering that RAGE inhibition can improve the immune environment, pharmacological inhibition of RAGE signaling was employed to assess neuronal status and cognitive function in mice (Figure 3A).

We first evaluated the effects of RAGE inhibition on hippocampal neurons. Nissl staining was performed on the hippocampus to assess neuronal numbers, and the data showed that the number of neurons was significantly restored in the RAGE inhibitor-treated group compared to the hypoxic group (Figure 3B,C). Golgi staining was performed on the hippocampus to assess synaptic changes, and the results showed that the RAGE inhibitor significantly restored the reduction in dendritic spine numbers caused by HAHH exposome (Figure 3D,E). To test whether RAGE inhibition is beneficial for improving cognitive impairment induced by HAHH exposome in mice, we established a HAHH-exposed mouse model and treated it with a RAGE inhibitor (RI group). To evaluate the effects of RAGE inhibition on cognitive and memory functions after HAHH exposome, we conducted Y-maze and novel object recognition tests (NORTs). The Y-maze test showed that the time spent exploring the novel arm was significantly shortened in the hypoxic group, while treatment with the RAGE inhibitor effectively improved the working memory impairment induced by HAHH exposome (Y-maze novel arm time: 28.08 ± 5.14 s vs. hypoxia 19.67 ± 4.68 s, *p* = 0.016) (Figure 3F,G). NORT data showed that the hypoxic group demonstrated preferential exploration of the novel object. Surprisingly, after treatment with the RAGE inhibitor, the recognition index and recognition time in the hypoxic group were almost reversed to normal levels (NORT recognition index: 0.52 ± 0.05 vs. hypoxia 0.33 ± 0.07, *p* < 0.001) (Figure 3H,I).

### 2.4. RAGE Inhibitors Reduce Microglial Inflammation via the MAPK Pathway and p65 Nuclear Translocation

**Figure 4:**
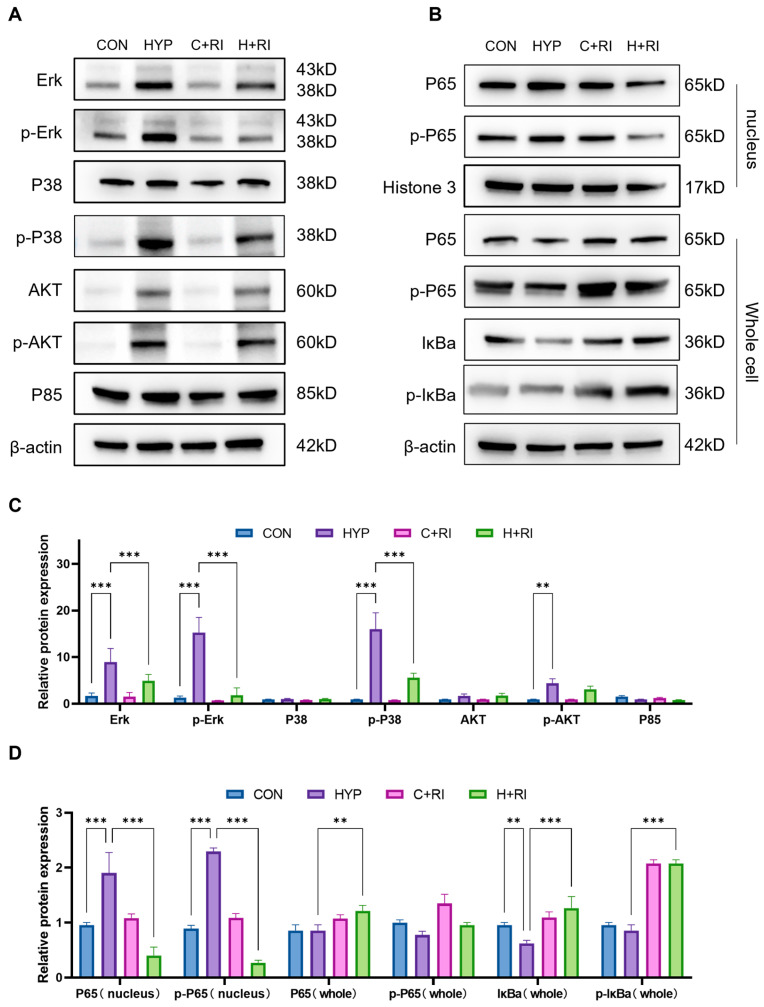
Mechanistic analysis of RAGE inhibitor on microglial inflammation via MAPK pathway. (**A**) WB analysis of MAPKs (ERK, P38, JNK) and PI3K/AKT phosphorylation in hippocampus of mice treated with RAGE inhibitor under hypoxic conditions. (**B**) WB analysis of NF-κB signaling pathway components (IκBα, p-IκBα, p65, p-p65) in hippocampus of mice treated with RAGE inhibitor under hypoxic conditions. (**C**) Quantitative analysis of (**A**), expressed as relative densitometry units normalized to total protein levels. (**D**) Quantitative analysis of (**B**). Data are presented as fold changes relative to control group. *n* = 3, analyzed by one-way ANOVA with Tukey post hoc test for multiple comparisons. ** *p* < 0.01, *** *p* < 0.001.

Next, we further explored the inflammatory molecular mechanisms of RAGE inhibition. RAGE triggers multiple downstream signaling cascades, notably the MAPK (ERK, p38, JNK) and PI3K/AKT pathways, whose activation promotes inflammatory reactions and oxidative stress, ultimately contributing to cellular and tissue injury. To assess the impact of RAGE inhibition on these pathways under hypoxic conditions, we measured the expression and phosphorylation levels of MAPKs (ERK, p38, JNK) and PI3K/AKT in the hippocampus of mice treated with the RAGE antagonist (Figure 4A). WB showed that the expression of p-ERK/P38/JNK and PI3K/AKT proteins was significantly increased in the hypoxic group by 9.1~15.2-fold compared to normoxic controls (CON; *p* < 0.01 to *p* < 0.001). However, the expression of p-ERK and p-p38 was significantly reduced by 81% and 49% when co-treated with the RAGE inhibitor relative to the HYP group (*p* < 0.01 to *p <* 0.001) (Figure 4C). The above data indicate that the MAPK pathway is involved in the protective effects of RAGE inhibitor, with ERK/P38 being the main signaling molecules. Compared to other receptor systems, RAGE exhibits unique Ras activation properties via MAPK signaling, thereby inhibiting the IκB cascade reaction and releasing and activating NF-κB. Therefore, we further detected the expression of NF-κB signaling pathway molecules at the protein level. Hypoxic conditions significantly elevated both IκBα and phosphorylated IκBα levels while increasing nuclear translocation of phosphorylated p65 (Figure 4B). However, when treated with FPS, the expression levels of p-IκBα and p-p65 in nuclear proteins were decreased, indicating reduced p65 nuclear translocation (Figure 4D). The above experiments confirm that the RAGE inhibitor inhibits microglial pro-inflammatory effects through the RAGE/MAPK/NF-κB pathway.

### 2.5. HAHH Exposome Upregulates RAGE Expression via HMGB1

To elucidate the molecular mechanisms underlying RAGE overexpression under hypoxic conditions, we screened and found that HMGB1, as a DAMP molecule, is upregulated under hypoxic conditions and can bind to RAGE to affect inflammatory and immune responses. These data lead us to propose HMGB1 as a potential upstream mediator of RAGE signaling in this system.

**Figure 5:**
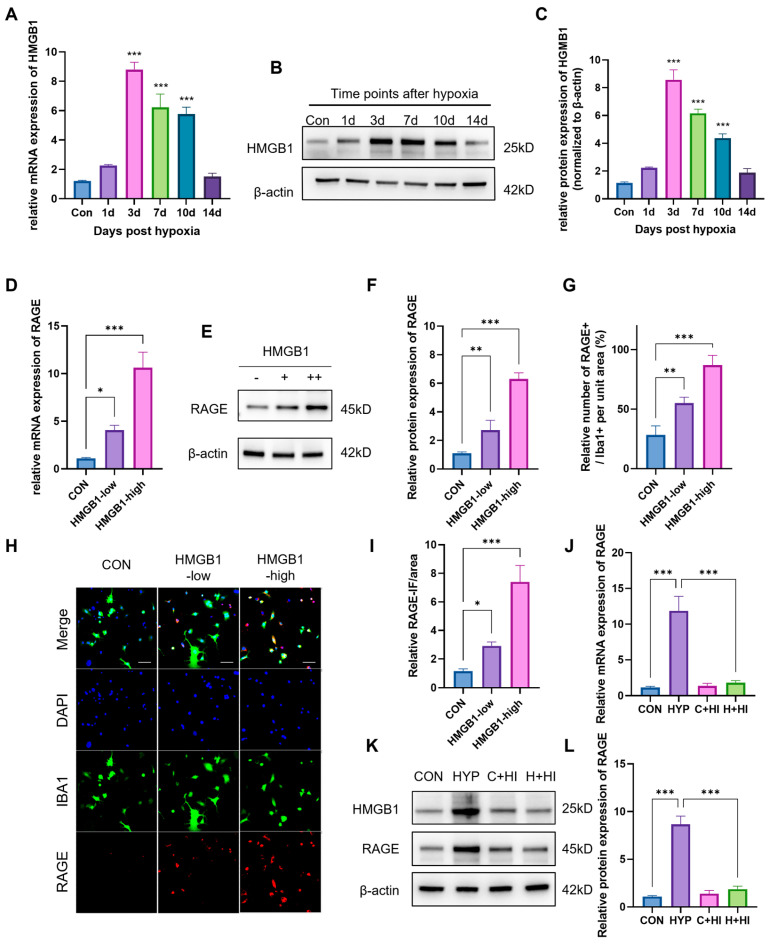
Regulation of RAGE expression by HMGB1 under hypoxic conditions. (**A**) qPCR analysis of HMGB1 mRNA levels in hippocampus of mice exposed to HAHH over time. Data are presented as fold changes relative to normoxic controls. (**B**) WB analysis of HMGB1 protein expression in hippocampus of mice exposed to HAHH over time. (**C**) Quantitative analysis of (**B**), normalized to β-actin. (**D**) qPCR analysis of RAGE mRNA levels in primary microglial cells treated with HMGB1 in vitro. (**E**) WB analysis of RAGE protein expression in primary microglial cells treated with HMGB1 in vitro. (**F**) Quantitative analysis of (**E**) expressed as relative densitometry units normalized to β-actin. (**G**) Quantitative analysis of RAGE-positive cells as percentage of IBA1-positive microglia in primary microglial cells treated with HMGB1 in vitro. (**H**) Representative images of staining for RAGE (green) and IBA1 (red) in primary microglial cells treated with HMGB1 in vitro. Scale bar = 20 μm. (**I**) Quantitative analysis of RAGE-positive cell proportion in primary microglial cells treated with HMGB1 in vitro. (**J**) qPCR analysis of RAGE mRNA levels in hippocampus of mice treated with HMGB1 inhibitor (glycyrrhizin) under hypoxic conditions. Data are presented as fold changes relative to hypoxic controls. (**K**) WB analysis of RAGE protein expression in hippocampus of mice treated with HMGB1 inhibitor under hypoxic conditions. (**L**) Quantitative analysis of (**K**). Data are presented as fold changes relative to control group. *n* = 3, analyzed by one-way ANOVA with Tukey post hoc test for multiple comparisons. * *p* < 0.05, ** *p* < 0.01, *** *p* < 0.001.

To verify the above hypothesis, we first detected the expression of HMGB1 under the HAHH exposome. qPCR data showed that the mRNA levels of HMGB1 significantly increased from 1 to 14 days, peaking at ~8.9-fold over normoxic controls (CON) by day 3 (*p* < 0.001 at 3, 7, 10 days timepoints) (Figure 5A). WB data also showed that HAHH exposome induced the expression of HMGB1 in the hippocampus, and the expression trend of HMGB1 was consistent with that of RAGE over time (Figure 5B,C).

To verify that HMGB1 regulates RAGE expression, we first treated microglial cells with HMGB1 in vitro and then detected RAGE expression using PCR and WB. HMGB1 treatment of microglia in vitro upregulated RAGE transcription ~10-fold vs. untreated cells (PCR: *p* < 0.001) and ~6.2-fold at the protein level (WB: *p* < 0.001) with high HMGB1 concentration (5 μM) (Figure 5D–F). Immunocytochemical data showed that after in vitro treatment with HMGB1, the immunofluorescence intensity (IFI) of RAGE (~4.5-fold, *p* < 0.001) and the number of RAGE^+^ cells in microglia significantly increased (~7.5-fold, *p* < 0.001) (Figure 5G–I). Subsequently, we used glycyrrhizin (an HMGB1 inhibitor) in mice and detected RAGE expression in primary microglia in vivo. PCR and WB results showed that use of the HMGB1 inhibitor reduced HMGB1 mRNA by 90% vs. hypoxic controls (*p* < 0.001), downregulated RAGE transcripts by 85% (*p* < 0.001) and decreased HMGB1 and RAGE protein expression by 75% (*p* < 0.001) (Figure 5J–L). This further proved that HMGB1 regulates RAGE expression under hypoxic conditions.

### 2.6. HMGB1-Induced Microglial Inflammation Under Hypoxic Conditions Can Be Reversed by RAGE Inhibitors

**Figure 6:**
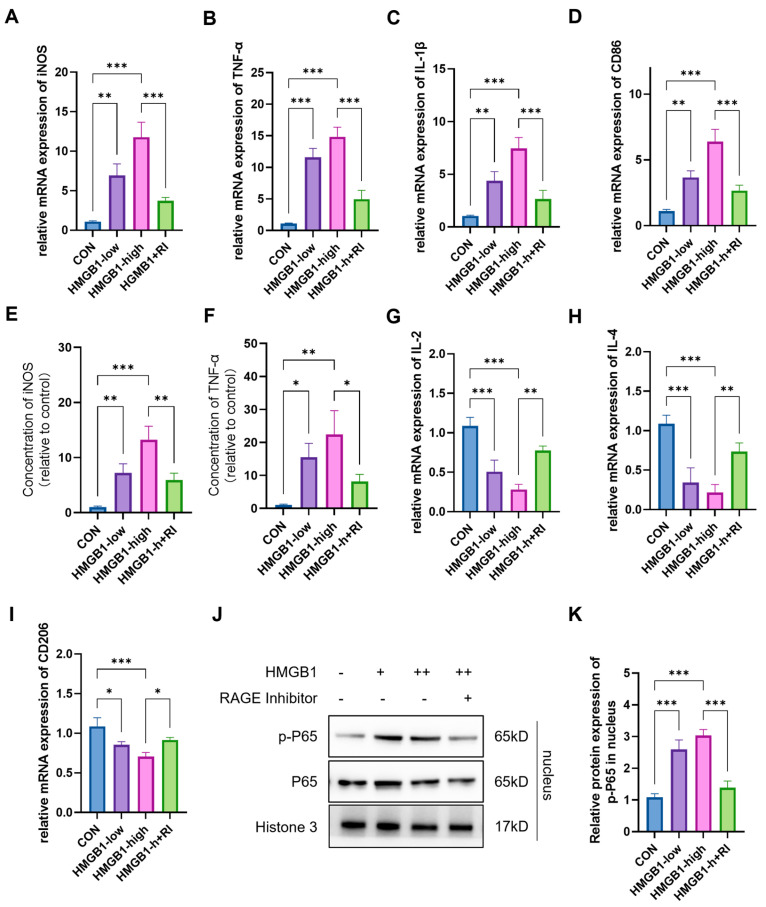
Effects of RAGE inhibitor on HMGB1-induced microglial inflammation. (**A**–**D**) qPCR analysis of iNOS, TNF-α, IL-1β, and CD86 mRNA levels in primary microglial cells treated with HMGB1 and RAGE inhibitor (FPS-ZM1). (**E**,**F**) ELISA analysis of iNOS and TNF-αlevels in primary microglial cells treated with HMGB1 and RAGE inhibitor. (**G**–**I**) qPCR analysis of IL-2, IL-4, and CD206 mRNA levels in primary microglial cells treated with HMGB1 and RAGE inhibitor. (**J**) WB analysis of p65 and p-p65 nuclear protein levels in primary microglial cells treated with HMGB1 and RAGE inhibitor. (**K**) Quantitative analysis of p65 and p-p65 nuclear protein levels in primary microglial cells treated with HMGB1 and RAGE inhibitor, expressed as relative densitometry units normalized to nucleus protein levels. Data are presented as fold changes relative to control group. *n* = 3, analyzed by one-way ANOVA with Tukey post hoc test for multiple comparisons. * *p* < 0.05, ** *p* < 0.01, *** *p* < 0.001.

To further delineate the mechanistic role of the HMGB1-RAGE axis in microglial inflammation under hypoxic conditions, pro- and anti-inflammatory gene expression profiles were compared between control and HMGB1-treated microglial cultures. The data showed that compared with cells under normal conditions, the mRNA levels of iNOS, TNF-α, IL-1β, and CD86 were significantly increased in microglial cells treated with HMGB1 (iNOS: ~12.5-fold; TNF-α: ~13.5-fold; IL-1β: ~7-fold; CD86: ~6-fold; *p* < 0.001 for all) (Figure 6A–F). A significant decrease was also observed in the mRNA levels of anti-inflammatory markers IL-2, IL-4, and CD206 after HMGB1 treatment (IL-2: ~0.3-fold; IL-4: ~0.3-fold; CD206: ~0.2-fold; *p* < 0.001), which was almost consistent with the changes observed following HAHH exposome (Figure 6G–I).

We further applied the RAGE-specific antagonist FPS-ZM1 to investigate whether HMGB1-triggered microglial polarization toward a pro-inflammatory phenotype is mediated through RAGE. The experimental data showed that the RAGE inhibitor significantly reversed the changes in mRNA levels of pro-inflammatory and anti-inflammatory markers induced by HMGB1. Specifically, RAGE inhibitor treatment significantly reduced the mRNA expression levels of HMGB1-induced pro-inflammatory markers (iNOS: 65%↓; TNF-α: 62%↓; IL-1β: 68%↓; CD86: 45%↓; *p* < 0.001) (Figure 6A–F) while upregulating the mRNA expression levels of anti-inflammatory markers (IL-2: 2.1-fold↑ vs. HMGB1; IL-4: 2.9-fold↑, *p* < 0.001; CD206: 1.2-fold↑, *p* < 0.05;) (Figure 6G–I). Additionally, WB data further confirmed that the RAGE inhibitor could significantly reduce the nuclear expression levels of the downstream molecule p-p65 of RAGE by ~65% (*p* < 0.001; vs. HMGB1) induced by HMGB1 (Figure 6J,K). These results indicate that the RAGE inhibitor effectively reversed HMGB1-triggered microglial polarization toward a pro-inflammatory phenotype by inhibiting RAGE and its downstream signaling pathway.

In summary, the above data lead us to conclude that the inflammatory response of microglia induced by the HAHH exposome is at least partially mediated by the HMGB1-RAGE-NF-κB signaling pathway. HMGB1 binds to RAGE, activating NF-κB-mediated transcriptional upregulation of pro-inflammatory cytokines and the exacerbation of inflammatory responses. The RAGE inhibitor, as a specific antagonist of RAGE, effectively inhibits the activation of this signaling pathway, thereby reducing the inflammatory response.

## 3. Discussion

Prolonged exposure to high-altitude hypoxic conditions leads to neurotoxicity, with the severity increasing with longer exposure durations [23,24,25]. The hypoxia exposome induces cognitive deficits through various mechanisms, including synaptic plasticity impairment, inflammation, apoptosis, mitochondrial dysfunction, and autophagy [26,27,28,29,30,31,32]. Microglia, as the immune cells of the CNS, play a key role in brain inflammation. Their overactivation results in the release of pro-inflammatory cytokines such as TNF-α and IL-1β, leading to neuronal apoptosis, axonal and synaptic damage, increased demyelination, and neurotoxicity [33,34,35]. Therefore, current pharmacological treatments for HAHH-induced neurotoxicity primarily focus on inhibiting overall inflammatory responses to alleviate symptoms. For instance, antioxidants and corticosteroids may improve cognitive function by reducing inflammation [36,37,38]. However, the mechanisms underlying microglial activation under hypoxic conditions remain incompletely understood. This study innovatively identified a novel mechanism of microglial activation and neurotoxicity under HAHH exposome, confirming that the HMGB1-RAGE-MAPK/NF-κB signaling pathway plays a crucial role in HAHH-induced microglial activation and inflammation. These data converge with evidence validating the core regulatory HMGB1-RAGE axis in inflammatory responses, which are involved in the release of inflammatory cytokines and the activation of inflammatory signaling pathways, and demonstrate substantial correlations with the emergence of various inflammatory diseases [20,21,22,39,40]. Our study further elucidates that HAHH exposome induces microglial inflammation via the HMGB1-RAGE-MAPK/NF-κB pathway, providing an experimental basis and theoretical support for the development of potential therapeutic strategies targeting these mechanisms.

RAGE activation plays a key role in various neurodegenerative diseases. RAGE is a multiligand inflammatory receptor that can bind to multiple ligands, including HMGB1, the S100 protein family, and amyloid-beta (Aβ), thereby influencing disease progression [41,42,43,44]. Validation studies are underway to assess its dual utility in diagnostic stratification and targeted intervention. For example, in Parkinson’s disease, RAGE expression is increased, and its binding to α-synuclein fibrils activates microglia, triggering inflammatory responses [45,46]. The interaction between RAGE and Aβ may exacerbate Alzheimer’s disease by increasing Aβ levels and activating inflammatory signaling pathways [47,48,49]. This study explored the changes in RAGE expression in hippocampal microglia under hypoxic conditions and its role in inflammation and cognitive impairment. We found that HAHH exposome significantly upregulated RAGE expression, activated microglia, and led to inflammation and cognitive deficits. Treatment with the RAGE inhibitor FPS-ZM1 significantly attenuated microglial inflammation and improved cognitive function in mice. Mechanistic studies revealed that the RAGE inhibitor reduced microglial inflammation by inhibiting the MAPK pathway and decreasing NF-κB activation. We confirmed HAHH induced pathway-selective MAPK activation: robust phosphorylation of p38/ERK with no JNK involvement. This branch-specific mechanism contrasts with uniform microglial activation in transient OGD/HIBD models and The subtype selectivity of downstream pathway redefines therapeutic optimization path of RAGE inhibition. Our findings suggest that RAGE inhibitors may serve as a novel therapeutic strategy with potential as a treatment target for mitigating HAHH-induced inflammation and cognitive impairment. It is worth noting that RAGE is expressed by neurons, glial cells, and endothelial cells, suggesting that RAGE inhibitors may exert their neuroprotective effects not only by directly inhibiting microglial inflammation but also through interactions with other cell types [50,51]. Therefore, we cannot rule out the possibility that RAGE inhibitors may have neuroprotective effects via mechanisms involving cells other than microglia.

HMGB1 is a key cytokine involved in various biological processes, particularly in inflammatory responses and cell signaling [52,53,54]. As a DAMP molecule, its expression is upregulated under hypoxic conditions, making it an important molecule in HAHH exposome research [54,55,56,57]. RAGE is a receptor for HMGB1, and their interaction activates multiple intracellular signaling pathways, influencing inflammatory and immune responses. The binding of HMGB1 to RAGE activates the phosphatidylinositol 3-kinase/protein kinase B (PI3K/AKT) and mitogen-activated protein kinase (MAPK) pathways, leading to the activation of nuclear factor-κB (NF-κB) and the subsequent release of cytokines such as TNF-α, IL-6, and IL-1α, as well as chemokines. This promotes inflammation, cell migration, apoptosis, and tissue remodeling [58,59,60,61,62]. Therefore, we hypothesized that HMGB1 acts upstream of RAGE. In this study, we confirmed that HMGB1 is an upstream regulator of RAGE, with its expression increasing under hypoxic conditions and positively regulating RAGE expression. Treatment with HMGB1 inhibitors reduced RAGE expression, thereby attenuating microglial inflammation and improving cognitive function. These results highlight the crucial role of the HMGB1-RAGE-MAPK/NF-κB signaling pathway in HAHH-induced inflammation, providing a potential therapeutic target. Additionally, studies have shown that HMGB1-induced RAGE expression creates a positive feedback loop that enhances HMGB1’s effects [22,39,63]. This suggests that under hypoxic conditions, the HMGB1-RAGE axis may be interconnected through various molecular mechanisms, and further research is needed to explore these interactions and develop potential therapeutic strategies.

While prior studies established HMGB1-RAGE involvement in acute hypoxic injury [9,10], unlike transient oxygen-glucose deprivation (OGD) or hypoxic–ischemic brain damage (HIBD) models, our severe high-altitude hypobaric hypoxia paradigm (6000 m equivalent) revealed how high-altitude hypobaric hypoxia alters learning and memory function by inducing microglial inflammation in hippocampus of mice via the HMGB1-RAGE axis and evaluate therapeutic inhibition strategies. The use of a multi-parameter high-altitude hypobaric hypoxia chamber (controlling O_2_, pressure, humidity, temperature) uniquely recapitulates environmental high-altitude stress, distinguishing it from OGD/HIBD models that simulate acute vascular events. While OGD/HIBD studies focus on immediate cell death pathways, HAHH reveals how chronic microglial inflammation (via HMGB1-RAGE) drives progressive cognitive impairment—a process more relevant to altitude-related neurodegeneration than stroke. This justifies the novelty of the therapeutic approach targeting sustained neuroinflammation. Unlike OGD/HIBD systems that induce acute normo-baric hypoxia, our model replicates the chronic synergistic stress driving altitude-induced neurodegeneration. Empirical evidence demonstrates that high-altitude hypoxia compromises core cognitive domains—specifically learning acquisition, memory consolidation, and executive reasoning [64,65,66,67]. Our laboratory previously validated this simulation chamber’s capacity to physiologically replicate high-altitude conditions using standardized murine exposure protocols [68,69].

While this research provides insights into prevention and treatment of cognitive impairment under HAHH, certain limitations merit attention. Female mice were excluded due to estrogen’s documented modulation of neuroinflammatory responses [70,71]. We exclusively used male mice in this study to eliminate confounding effects of estrogen fluctuation on neuroinflammatory responses, avoid hormonal cycle variations, and maintain consistency with established hypoxia research models. While male-only use controls for hormonal confounders, future studies should evaluate sex-specific responses to hypoxia. Although HMGB1-RAGE signaling constitutes the primary driver of HAHH-induced neuroinflammation, studies suggests that compensatory TLR4 engagement [49,72,73] and NLRP3 inflammasome crosstalk [74,75] may also serve as secondary function. Critically, astrocyte-derived HMGB1 may amplify this cascade [18,76]—a paracrine mechanism that warrants validation in future neuron–microglia–astrocyte tri-culture studies. Also, the current lack of neuron-microglia co-culture data represents a limitation; to address this gap, in vitro co-culture systems examining bidirectional signaling are currently in progress.

## 4. Materials and Methods

### 4.1. Hypoxic Exposure and Animal Models

Male C57BL/6 mice (6–8 weeks old) were used to standardize neurodevelopmental status and eliminate estrogen-mediated variability. Mice were housed in the specific pathogen-free (SPF) animal facilities in a climate controlled clean room with humidity range of 40–70% and temperature range of 20–26 °C, with a 12 h light/dark cycle and fed with regular irradiated maintenance diet for mice (MD17121, medicience) containing 20% crude protein, 6% fat, and 5% fiber, with Co60 irradiation for pathogen elimination and water throughout the study. A multi-environment simulation chamber (Guizhou Fenglei, Guiyang, China) was selected to simultaneously replicate real-world hypoxic conditions at high altitude by controlling oxygen (10.5%), temperature (22 ± 2 °C), humidity (55 ± 5%) and atmospheric pressure. This severity was classified as severe hypoxia and selected for inducing significant neuropathology (cognitive deficits, neuroinflammation) while maintaining animal viability (100% survival) and representing a pathologically relevant high-altitude environment for mechanistic studies [68,69]. Mice were randomly grouped in groups (normoxia/hypoxia/vehicle/drug) via computer-generated sequences. The hypoxia group was exposed to the simulated high-altitude environment for the specified duration, while the normoxic group was maintained under standard laboratory conditions throughout the experiment. Two leveraging advanced inhibitors were used for axis blocking: FPS-ZM1 (10 mg/kg/day i.p. in vivo; 10 μM in vitro), a specific RAGE antagonist that binds the ligand-binding domain [77] and Glycyrrhizin (20 mg/kg/day i.p.), an established HMGB1 inhibitor that blocks receptor binding [15,78]. All procedures strictly adhered to the principles delineated in the Declaration of “Guiding Principles in the Care and Use of Animals” (China). This study was conducted in accordance with the animal protocol approved by the Institutional Animal Care and Use Committee (Air Force Medical University, Xi’an, China).

### 4.2. RNA Sequencing and Bioinformatics Analysis

Total RNA was isolated from mouse hippocampal tissues using TRIzol reagent (Invitrogen, Waltham, MA, USA) as instructed. Gene Denovo (Shenzhen, China) conducted RNA sequencing. Genes with differential expression were identified when the *p* value < 0.05 and the fold change > 1.2. Gene ontology (GO) and Kyoto Encyclopedia of Genes and Genomes (KEGG) pathway enrichment analyses were performed.

### 4.3. Quantitative Real-Time PCR (qPCR)

Hippocampal tissue samples were homogenized in TRIzol reagent (Invitrogen) for total RNA extraction. Reverse transcription to generate cDNA was carried out with the PrimeScript RT Kit (Takara, Tokyo, Japan). Amplification reactions were monitored with the TB Green Premix Ex Taq II FAST qPCR (Takara, Japan) on a QuantStudio 7 Real-Time PCR System (Applied Biosystems, Waltham, MA, USA). Relative quantification of target genes, normalized against β-actin as the endogenous control, was performed using the 2^−ΔΔCt^ algorithm. Primer sequences are provided in Supplementary Table S1.

### 4.4. WB Analysis

Protein from hippocampal tissue homogenates and primary microglial cell lysates were prepared using RIPA lysis buffer (Servicebio, Wuhan, China) containing protease and phosphatase inhibitor cocktails. Quantification of protein concentrations was determined via bicinchoninic acid assay (BCA Kit, Servicebio, China) following the manufacturer’s protocols with samples normalized to 2 μg/μL before SDS-PAGE. Electrophoretic separation of equal protein quantities (30–50 μg/lane) was conducted on 10–12% SDS-polyacrylamide gels followed by semi-dry transfer onto PVDF membranes (Millipore, Billerica, MA, USA). Membrane blocking was achieved through 1 h incubation with 5% skimmed milk dissolved in Tris-buffered saline containing 0.1% Tween-20 (TBST). Primary antibody incubations were performed at 4 °C for 12–16 h using the following reagents: rabbit monoclonal anti-RAGE (Abcam, Cambridge, MA, USA, ab3611, 1:1000), rabbit polyclonal anti-HMGB1 (Abcam ab18251, 1:1000), rabbit monoclonal anti-p65 (CST #6956, 1:1000), rabbit monoclonal anti-phospho-p65 (Ser536, CST, Danvers, MA, USA, #3033, 1:1000), and mouse monoclonal β-actin (Abcam ab6276, 1:5000) as the loading control. After TBST washes (3 × 10 min), membranes were exposed to species-matched HRP-conjugated secondary antibodies (Servicebio, 1:5000) for 60 min at ambient temperature. Chemiluminescent signals were generated using an enhanced ECL substrate (Servicebio) and captured digitally. Densitometric analysis was conducted using ImageJ V1.8.0 software (NIH, Bethesda, MD, USA) with β-actin normalization for quantitative comparisons.

### 4.5. Immunohistochemistry and Immunofluorescence Staining

Mice were heavily anesthetized, then their hearts were first flushed with 0.9% saline and then 4% paraformaldehyde. Their brains were taken out and fixed in 4% paraformaldehyde for 24 h, after which they were moved to a 30% sucrose solution to protect them from freezing. Sections (thin slices) of the brain were first blocked using 5% normal goat serum in PBS (phosphate-buffered saline) for an hour at room temperature. Then, they were mixed with primary antibodies and left overnight at 4 °C. After washing away excess antibodies with PBS, the sections were incubated with biotinylated secondary antibodies for an hour at room temperature. DAPI was used to stain the nuclei of cells. Finally, images were taken using a fluorescence microscope made by Olympus in Japan. The primary antibodies used in this study were anti-RAGE (1:200, Abcam, Cambridge, MA, USA, ab3611) and anti-HMGB1 (1:200, Abcam, ab18251).

### 4.6. Primary Microglial Cell Culture and Treatment

Brains were dissected and dissociated into single-cell suspensions. Cells were plated in DMEM/F12 medium (Gibco, Waltham, MA, USA) with 10% fetal bovine serum and cultured at 37 °C in 5% CO_2_. After 10–14 days, microglial cells were purified by shaking off oligodendrocyte precursor cells and astrocytes. For hypoxia treatment, cells were placed in the hypoxia chamber (Don Whitley, Bingley, UK) with 1% O_2_, 5% CO_2_, and 94% N_2_ for the specified duration. For HMGB1 treatment, cells were stimulated with recombinant mouse HMGB1 (100–500 ng/mL, R&D Systems, Minneapolis, MN, USA) for 24 h. For RAGE inhibitor treatment, cells were pre-treated with FPS-ZM1 (10 μM, Tocris, Bristol, UK) for 1 h before hypoxia or HMGB1 treatment.

### 4.7. Flow Cytometry Analysis

Microglial cells were collected and suspended in PBS with 2% FBS. They were then stained with the following antibodies for 30 min at 4 °C: anti-CD86-PE (1:100, BD Biosciences, San Jose, CA, USA, 553690) and anti-CD206-FITC (1:100, BD Biosciences, 556049). After PBS washing, the cells were analyzed via flow cytometry.

### 4.8. Cognitive Function Tests

#### 4.8.1. Y-Maze Test

In the Y-maze test for assessing mice’s working memory [79], the maze has three same-sized arms (40 cm × 12 cm × 15 cm) set at 120° to each other. Mice begin at the start arm’s end, exploring two open arms for 5 min with the novel arm closed. After the first time interval, they again start at the end of the start arm, this time exploring all three open arms for 5 min. A video tracking system (Xinruan, Shanghai, China) records the time mice spent in each arm. Behavioral videos were analyzed by blinded investigators using coded IDs. This percentage is determined by using the following formula: (time in novel arm/total time in all arms) × 100.

#### 4.8.2. Novel Object Recognition Test (NORT)

In the NORT for assessing mice’s recognition memory [80], there are two phases: training and testing. During training, mice are placed in a square box and explore two identical objects for 5 min. Then, in testing, they are put back in the box for 5 min with one familiar object and one novel object. A video tracking system (Xinruan, Shanghai, China) records the exploration time for each object. Behavioral videos were analyzed by blinded investigators using coded IDs. The recognition index is calculated as follows: (time exploring novel object/total exploration time for both objects) × 100.

### 4.9. Statistical Analysis

Data are shown as means ± SD. For statistical analysis, we used one-way ANOVA with Tukey post hoc test for multiple comparisons or Student’s *t*-test for two-group comparisons. A *p*-value < 0.05 was seen as statistically significant. ns, *p* > 0.05, ** *p* < 0.01, *** *p* < 0.001. All analyses were performed with GraphPad Prism software (GraphPad 9.3, La Jolla, CA, USA). Molecular/histological quantitation (WB/IHC/flow cytometry) used anonymized samples processed by independent blinded personnel via ImageJ V1.8.0 software (NIH, Bethesda, MD, USA).

All behavioral data were included only from animals that completed >90% of the test duration (≥270 s for both Y-maze and NORT 5 min sessions), with NORT datasets requiring ≥20 s of cumulative object exploration. Western blot quantification required bands to fall within the linear detection range, as verified by β-actin loading controls. Immunofluorescence analysis mandated examination of ≥3 fields per tissue section at 20× magnification. Predefined health abnormalities triggered exclusion: weight loss exceeding 20% baseline or spontaneous seizure occurrence at any experimental stage. Molecular datasets were discarded for RNA degradation (28S:18S ribosomal ratio < 1.8). IHC staining failures evidenced by negative control aberrations (over-staining or under-staining).

## 5. Conclusions

In summary, this study identifies key mechanisms by which the RAGE pathway modulates microglial responses during HAHH, suggesting that targeted inhibition of HMGB1–RAGE signaling may attenuate HAHH-associated inflammation and cognitive deficits. These preclinical findings highlight the potential of HMGB1–RAGE axis modulation as a therapeutic strategy, though translational applications require further validation across diverse hypoxic contexts and disease models. Future work should prioritize defining context-specific roles of HMGB1–RAGE in HAHH versus acute hypoxia paradigms, establishing robust biomarkers for patient stratification, and evaluating clinically viable inhibitors to bridge these mechanistic insights toward practical neuroprotection.

## Data Availability

The data used to support the findings of this study are available from the corresponding author upon request.

## Supplementary Materials

The following supporting information can be downloaded at: https://www.mdpi.com/article/10.3390/ijms26188782/s1.

## Abbreviations

The following abbreviations are used in this manuscript:

RAGE	Receptor for Advanced Glycation End-Products
HMGB1	High-Mobility Group Box 1
CNS	Central Nervous System
DAMP	Damage-Associated Molecular Pattern
MAPK	Mitogen-Activated Protein Kinase
PI3K	Phosphatidylinositol 3-Kinase
AKT	AKT Serine/Threonine Kinase
JNK	c-Jun N-terminal Kinase
ERK	Extracellular Signal-Regulated Kinase
